# Chemical, genetic and structural assessment of pyridoxal kinase as a drug target in the African trypanosome

**DOI:** 10.1111/j.1365-2958.2012.08189.x

**Published:** 2012-08-16

**Authors:** Deuan C Jones, Magnus S Alphey, Susan Wyllie, Alan H Fairlamb

**Affiliations:** Division of Biological Chemistry & Drug Discovery, College of Life Sciences, University of DundeeDundee, UK

## Abstract

Pyridoxal-5′-phosphate (vitamin B_6_) is an essential cofactor for many important enzymatic reactions such as transamination and decarboxylation. African trypanosomes are unable to synthesise vitamin B_6_*de novo* and rely on uptake of B_6_ vitamers such as pyridoxal and pyridoxamine from their hosts, which are subsequently phosphorylated by pyridoxal kinase (PdxK). A conditional null mutant of PdxK was generated in *Trypanosoma brucei* bloodstream forms showing that this enzyme is essential for growth of the parasite *in vitro* and for infectivity in mice. Activity of recombinant *T. brucei* PdxK was comparable to previously published work having a specific activity of 327 ± 13 mU mg^−1^ and a *K*_m_^app^ with respect to pyridoxal of 29.6 ± 3.9 µM. A coupled assay was developed demonstrating that the enzyme has equivalent catalytic efficiency with pyridoxal, pyridoxamine and pyridoxine, and that ginkgotoxin is an effective pseudo substrate. A high resolution structure of PdxK in complex with ATP revealed important structural differences with the human enzyme. These findings suggest that pyridoxal kinase is an essential and druggable target that could lead to much needed alternative treatments for this devastating disease.

## Introduction

Two subspecies of *Trypanosoma brucei* (*T. b. gambiense* and *T. b. rhodesiense*) are responsible for human African trypanosomiasis, a parasitic disease endemic to sub-Saharan Africa. The estimated population at risk is 50 million, with around 30 000 deaths per year (Stuart *et al*., 2008). Treatment for African sleeping sickness has improved somewhat following the introduction of nifurtimox–eflornithine combination therapy (Yun *et al*., 2010) for the late stage of the disease. However, all available treatments remain unsatisfactory due to factors such as the need for parenteral administration, increasing treatment failures, possibly due to drug resistance, drug toxicity and cost (Burri, 2010). Hence there remains an urgent need for the discovery of new drug targets and the development of new drugs.

Sequencing the *T. brucei* genome has greatly enhanced our ability to identify potential drug targets (Berriman *et al*., 2005). However, it is imperative to assess them for essentiality and druggability according to a number of criteria (Frearson *et al*., 2007; Wyatt *et al*., 2011). These include target validation, assay feasibility for compound screening, potential resistance mechanisms, potential host toxicity and structural information. Another attractive feature of a potential drug target is one that causes pleiotropic downstream effects that are rapidly cidal rather than cytostatic.

Pyridoxal-5′-phosphate (PLP, a B_6_ vitamer) is an essential and versatile cofactor for a wide range of enzyme reactions, including transamination, decarboxylation, racemization and elimination or replacement reactions of amino acids (Eliot and Kirsch, 2004). Vitamin B_6_ has also been implicated as an antioxidant in resistance to singlet oxygen (^1^O_2_) (Ehrenshaft *et al*., 1999), a reactive oxygen species produced by photochemical or enzyme-mediated reactions such as the respiratory burst (Ziegelhoffer and Donohue, 2009; Cadet *et al*., 2010). Plants, fungi and some microorganisms [e.g. *Mycobacterium tuberculosis* (Dick *et al*., 2010) and *Plasmodium falciparum* (Wrenger *et al*., 2008)] can synthesise vitamin B_6_*de novo* either from deoxyxylulose 5-phosphate and 4-phosphohydroxy-l-threonine or from ribulose 5-phosphate, dihydroxyacetone phosphate and glutamine. In contrast, other organisms, including humans, salvage pyridoxal from their diet (Fitzpatrick *et al*., 2010). In the salvage pathway, extracellular PLP is first converted to pyridoxal (PL) by non-specific alkaline phosphatases, then PL or the B_6_ vitamers, pyridoxine (PN) and pyridoxamine (PM), are taken up into cells where they are phosphorylated by the enzyme pyridoxal kinase (PdxK). PNP and PMP are subsequently converted to the biologically relevant form PLP.

Genome analysis suggests that trypanosomatids, including the African trypanosome *T. brucei*, lack genes for *de novo* biosynthesis of vitamin B_6_ and are therefore dependent on the salvage pathway, particularly PdxK, for the production of PLP. This corresponds with the observation that pyridoxamine or pyridoxal, but not pyridoxine, is essential for growth in fully defined medium of the related insect trypanosomatid *Crithidia fasciculata* (Kidder and Dutta, 1958). Given the extensive use of PLP as a cofactor, removal or inhibition of PdxK activity should therefore result in multiple metabolic catastrophes within the parasite. Indeed, whole genome analysis of *T. brucei* at the B_6_ Database (http://bioinformatics.unipr.it/B6db) (Percudani and Peracchi, 2009) identified 25 genes with pyridoxal binding sites, of which 16 could be assigned to Enzyme Commission numbers. These included amino acid transferases, enzymes of folate metabolism (glycine dehydrogenase component of the glycine cleavage system) and polyamine biosynthesis (ornithine decarboxylase).

Human cells also depend on the salvage pathway for production of PLP. Although dietary deficiency of vitamin B_6_ is rare, there are several inborn errors of metabolism that can result in peripheral neuropathy or seizures (Clayton, 2006). Certain drugs can also react with and sequester PLP. For example, chronic administration of isoniazid for the treatment of tuberculosis can result in peripheral neuropathy and acute isoniazid overdose can induce convulsions (Snider, 1980; Watkins *et al*., 1990). However, drug-induced toxicity is readily treated with vitamin B_6_. The natural product ginkgotoxin, which is a component in *Ginkgo biloba* leaves used in teas and in seeds eaten in Asian countries, is a potent neurotoxin. Ginkgotoxin is a competitive substrate for pyridoxal kinase and can cause seizures, possibly by depleting the brain of phosphorylated B_6_ vitamers required for neurotransmitter metabolism (Leistner and Drewke, 2010). Thus, lack of selectivity between the human and *T. brucei* PdxK will be a key potential toxicity issue in downstream drug development.

Some of the properties of recombinant *T. brucei* pyridoxal kinase have been reported previously (Scott and Phillips, 1997). In the present study, the kinetic characterization of the enzyme is extended; essentiality *in vitro* and *in vivo* is demonstrated; and the crystal structure of the enzyme solved and key differences in the active site identified with the human enzyme. As a result, this enzyme is now suitable for entry into the drug discovery pipeline.

## Results and discussion

### Generation of a *TbPdxK* conditional null mutant

Restriction analysis of genomic DNA from *T. brucei* and subsequent Southern blotting using a probe to the full open reading frame (ORF) confirmed that *PdxK* is single-copy per haploid genome (data not shown). To create a conditional *TbPdxK* null mutant (conditional double knockout; cDKO), knockout constructs were prepared by PCR (see primers in Table S1) in which drug resistance markers (puromycin *N*-acetyl transferase, *PAC,* or hygromycin phosphotransferase, *HYG*) were inserted between the 5′- and 3′-untranslated regions flanking the ORF of *TbPdxK.* The first allelic copy of *TbPdxK* was replaced by transfection with the *PAC* construct followed by selection with puromycin. A tetracycline-inducible copy of *PdxK* was then inserted into the rDNA locus using a pLew100 construct conferring resistance to phleomycin. Tetracycline was added to cell cultures to induce expression of *Tb*PdxK in the rDNA locus before transfection with the *HYG* knockout construct. Southern blot analysis of genomic DNA from each stage of this process confirmed that the two *TbPdxK* alleles had been replaced with *PAC* and *HYG* and that a rescue copy had been correctly targeted to the rDNA locus (not shown).

### Effect of vitamin B_6_ on growth

The cDKO demonstrated no obvious growth defect or gross morphological changes when cultured in HMI9T medium in the presence of tetracycline. However, removal of tetracycline from the medium resulted in growth of cells slowing or arresting around day 5 (Fig. S1). Growth resumed within 2–3 days, a common observation in this system as conditional null mutants lose tetracycline-control by a variety of mechanisms (Roper *et al*., 2002).

We reasoned that even a low basal level of non-induced expression in the cDKO might be sufficient to partially mask the essential requirement for *Tb*PdxK, particularly if the intracellular vitamer substrate concentration was saturating. This led us to estimate the vitamin B_6_ content of HMI9T medium. Levels of vitamin B_6_ in human and fetal calf serum are not significantly different at around 200 nM (Baker *et al*., 1988), with the non-phosphorylated vitamers being approximately 50 nM in human serum (Gray, 1995). In contrast, the total concentration of non-phosphorylated vitamers in HMI9T was calculated to be 16.9 µM as follows: PN present in 80% IMDM medium (15.5 µM); PL present in the 10% Serum Plus culture additive (1.34 µM – SAFC Biosciences, pers. comm.); and various vitamers present in 10% FCS (approximately 20 nM). Vitamin B_6_ in HMI9T is therefore approximately 340-fold higher than physiological levels in serum. The cDKO was therefore cultured in vitamin B_6_-deficient medium (PDM) based on HMI9T, wherein 10% Serum Plus was replaced with water and the IMDM was assembled in-house without vitamin B_6_.

**Fig. 1:**
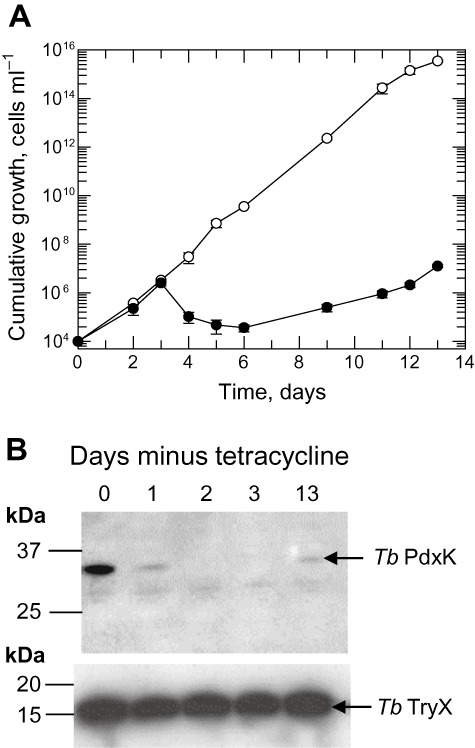
Growth characteristics and biochemical analysis of cDKO cells *in vitro*. A. The growth of the cDKO cell line in PDM was assessed in the presence (open circles) and absence of tetracycline (closed circles). B. Expression of PdxK in cDKO cells was assessed by immunoblotting at various points in the time-course. Trypanosomes (5 × 10^6^ per lane) were probed with antiserum to *T. brucei* PdxK and to *T. brucei* TryX as a control.

The cDKO trypanosomes cultured in PDM plus tetracycline for 6 months showed no apparent growth deficiency or gross morphological defects. However, removal of tetracycline from the medium resulted in cell death by day 4 (Fig. 1A). Western blotting using antiserum raised against recombinant *Tb*PdxK showed that the majority of *Tb*PdxK had been lost by day 1 and was undetectable by day 2 (Fig. 1B). As noted for B_6_-rich medium, growth resumed within 4–5 days, and these ‘revertant’ cells now resumed expression of PdxK (Fig. 1B).

Given the ameliorating effects of high vitamin B_6_ in HMI9T, we compared the effects of high (1.34 µM) concentrations of various vitamers on the growth of the cDKO line in PDM in the absence of tetracycline. Both PL and PM, but not PN, attenuated the effect of PdxK depletion allowing greater cell growth (Fig. S2). This led us to conclude that the trypanosomes are not able to convert PN to PLP. Putrescine failed to protect the cDKO from cell death in the absence of tetracycline, thus the essential requirement for *Tb*PdxK was not solely the result of loss of ornithine decarboxylase activity, a PLP-dependent enzyme, essential for polyamine and trypanothione biosynthesis (Fairlamb *et al*., 1987; Li *et al*., 1998).

### Virulence in mice

**Fig. 2:**
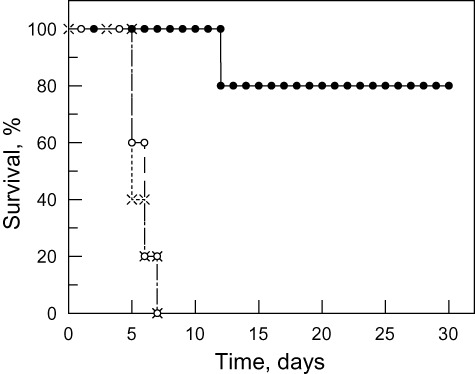
Virulence of WT and cDKO *T. brucei* infections in mice. Results are presented as a Kaplan–Meier survival plot. Symbols: WT cells (×) and cDKO with (•) or without (○) doxycycline in drinking water.

Due to the differences between *in vitro* culture and the *in vivo* environment, where possible, essentiality of drug targets should be assessed in animal models (Frearson *et al*., 2007; Wyatt *et al*., 2011). Groups of mice were infected with wild-type (WT) or cDKO *T. brucei* and the course of the infection monitored over a 30-day period (Fig. 2). Mice provided with doxycycline in their drinking water and infected with cDKO trypanosomes survived approximately 5 days, as did those infected with WT cells. However, four out of five cDKO-infected mice without doxycycline induction remained completely free of parasites and survived beyond 30 days while the fifth mouse in this group succumbed to infection on day 12; this was most likely due to a loss of tetracycline control in these parasites, a common phenomenon in conditional null mutants of essential genes in *T. brucei* (Roper *et al*., 2002; Wyllie *et al*., 2009). These results demonstrate that *Tb*PdxK is absolutely essential for parasite survival under the physiological conditions present in the host.

### Cloning, expression and purification of recombinant *Tb*PdxK

**Fig. 3:**
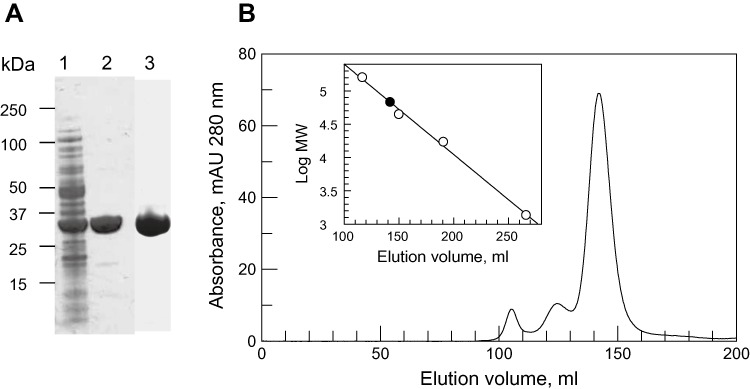
Recombinant expression of *T. brucei* PdxK. *Tb*PdxK was expressed from pET3a in BL21 (DE3) cells. A. SDS-PAGE analysis, 10 µg protein was loaded per lane. Lane 1, clarified lysate; lane 2, fractions pooled from the Q-sepharose column; lane 3, fractions pooled from gel filtration. B. Gel filtration of Q-sepharose-purified *Tb*PdxK. The inset shows a plot of elution volume vs. log molecular mass of a mixture of protein standards. The closed circle represents the elution volume of *Tb*PdxK.

Sequencing of the 903 bp ORF revealed a single difference from the previously published sequence (Scott and Phillips, 1997) (Accession number U96712), where guanine at position 861 was replaced with adenine in all three independent PCR products; however, this point mutation does not affect the amino acid sequence. Following induction with isopropyl-β-thio-d-galactoside (IPTG), *Escherichia coli* BL21 strain that had been transformed with a pET3a-PdxK construct expressed a prominent band around the expected molecular weight of 33 kDa in the soluble protein fraction (Fig. 3A, lane 1). The protein was purified by ion exchange chromatography with a yield of 125 mg l^−1^ of culture (Fig. 3A, lane 2) and its identity confirmed by peptide mass fingerprinting. Gel filtration indicated that greater than 90% of the protein eluted as a dimer (Fig. 3B) and removed some minor contaminants (Fig. 3A, lane 3). However, the protein obtained after ion exchange was of sufficient purity for kinetic characterization, raising antibodies and crystallography.

### Kinetic characterization by direct assay (388 nm)

The *T. brucei* enzyme has been previously assayed in 70 mM potassium phosphate pH 7.0, 150 µM ZnCl_2_, 1.5 mM ATP and 300 µM PL at 37°C by the increase in absorbance at 388 nm (Scott and Phillips, 1997). The specific activity for the native enzyme was 520 mU mg^−1^ with no value reported for the recombinant enzyme. The reported *K*_m_^app^ with respect to PL for the recombinant enzyme was 38 ± 6.4 µM. Under identical assay conditions our recombinant enzyme compares well with a specific activity of 327 ± 13 mU mg^−1^. Fitting the data to the Michaelis–Menten equation gave a *K*_m_^app^ with respect to PL of 29.6 ± 3.9 µM.

**Fig. 4:**
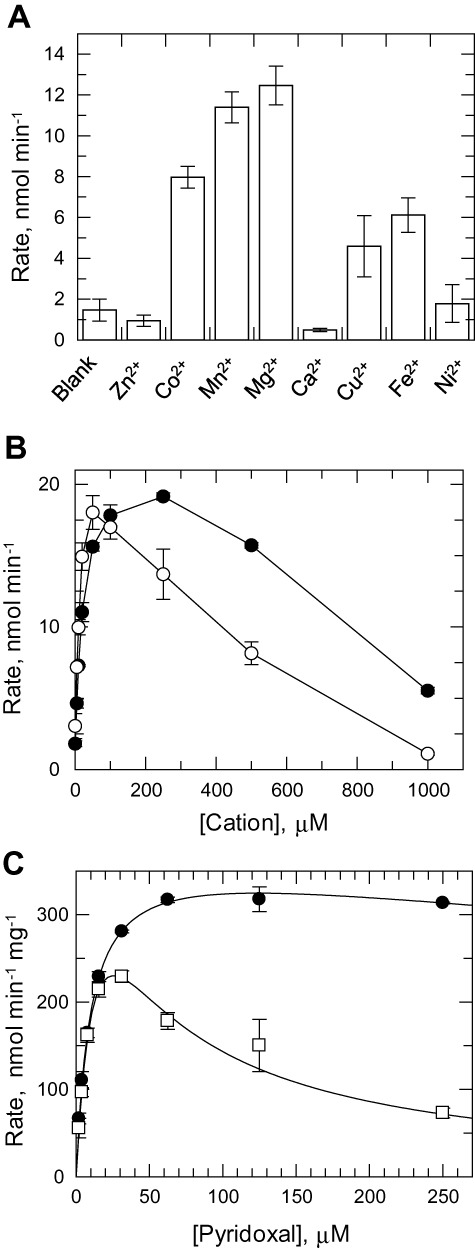
Assay optimization. PdxK activity was measured by increase in absorbance at 388 nm resulting from formation of PLP in 100 mM HEPES buffer, pH 7.4 and 1.5 mM ATP. A. Divalent cations were tested at 100 µM in the presence of 300 µM pyridoxal in duplicate. B. The two cations conferring the highest activity (MgCl_2_ and MnCl_2_) were titrated from 1 mM to 5 µM in duplicate. C. Kinetic behaviour of the enzyme was determined in the presence of 250 µM MgCl_2_ (closed circles) or 150 µM ZnCl_2_ (open squares). Data were fitted to the equation describing high substrate inhibition as described in the experimental procedures.

Further optimization of the assay was carried out in 100 mM HEPES buffer at pH 7.4 (physiological pH) and at room temperature (27°C; convenient for high throughput screening). Monovalent and divalent metal cations are required for ATP binding and substrate catalysis in many kinases (Di Cera, 2006), so a variety of divalent metal cations were screened at 100 µM. Under these conditions ZnCl_2_ did not activate the enzyme, whereas MgCl_2_ and MnCl_2_ were most active (Fig. 4A). These two cations were then titrated from 5–1000 µM, reducing PL to 200 µM since in pilot *K*_m_ determinations this concentration gave no substrate inhibition. MnCl_2_ gave a sharp optimum activity at 50 µM, whereas MgCl_2_ had a broader peak centred on 250 µM (Fig. 4B). The optimum with MgCl_2_ did not change significantly when carried out at 5 µM PL (data not shown). Initial kinetic studies comparing 150 µM ZnCl_2_ with 250 µM MgCl_2_ in our standard assay conditions (Fig. 4C) revealed that ZnCl_2_ showed pronounced high substrate inhibition (*K*_i_^s^ 39.8 ± 4.8 µM) compared with MgCl_2_ (*K*_i_^s^ 1540 ± 130 µM). This inhibitory effect of ZnCl_2_ accounts for the apparent lack of activation in Fig. 4A. The ability of phosphate to form complexes with divalent metal ions is consistent with the less pronounced inhibition observed in phosphate (*K*_i_^s^ 174 ± 23 µM) compared with HEPES buffer. With a new preparation of recombinant enzyme, the resulting kinetic constants for our optimized assay conditions in HEPES/MgCl_2_ pH 7.4 were *k*_cat_ 131 ± 2 s^−1^; *K_m_^app^* 1.65 ± 0.14 µM; *K*_i_^s^ 530 ± 56 µM; *k*_cat_/*K*_m_^app^ 9.0 × 10^7^ M^−1^ s^−1^ (weighted mean and weighted standard deviation of three independent determinations).

### Kinetic characterization by coupled assay (340 nm)

The direct spectrophotometric assay relies on the marked difference in absorption coefficient at 388 nm between PL and PLP (Table S2). However, this method is not suitable for assaying other B_6_ vitamers. We therefore developed a coupled assay where formation of the product ADP was coupled to consumption of NADH using pyruvate kinase and lactate dehydrogenase. In the case of PL and PM, Δ absorption coefficients at 340 nm (Table S2) were determined to correct for the increased absorbance of the phosphorylated vitamers. In the case of GT and PN, the increase in absorbance at 340 nm was negligible.

Under the coupled assay conditions, recombinant *Tb*PdxK had a specific activity of 57.7 ± 4.6 mU mg^−1^, which is sixfold less than in the direct assay. This lower activity is most likely due to the higher concentration of Mg^2+^ required for activity of the coupling enzymes (1 mM) as this concentration was shown to be inhibitory in the direct assay (Fig. 4B). Kinetic parameters for PL, PN and PM are given in Table 1 where the *k*_cat_/*K*_m_^app^ shows that the enzyme has equivalent catalytic efficiency with each of these vitamers. In addition, ginkgotoxin is also an effective pseudo-substrate for *Tb*PdxK.

**Table 1:** Kinetic parameters for TbPdxK with B_6_ vitamers and ginkgotoxin

Substrate	*K*_m_^app^ (µM)	*k*_cat_ (s^−1^)	*k*_cat_/*K*_m_^app^ (M^−1^ s^−1^)
Pyridoxal	14.8 ± 0.7	27.3 ± 1.3	1.84 × 10^7^
Pyridoxine	48.0 ± 12.7	122 ± 7	2.54 × 10^7^
Pyridoxamine	59.4 ± 14.8	127 ± 15	2,14 × 10^7^
Ginkgotoxin	46.6 ± 10.2	106 ± 2	2.28 × 10^7^

Details of the coupled assay system are described in *Experimental procedures*.

### Effect of ginkgotoxin and other PdxK inhibitors on *T. brucei*

Three reported inhibitors of PdxK were tested against *T. brucei* bloodstream forms using a resazurin-based assay of cell proliferation (Raz *et al*., 1997; Jones *et al*., 2010). 4-Deoxypyridoxine, reported to be an inhibitor of porcine PdxK competitive with respect to PL (Kwok and Churchich, 1979), failed to inhibit *T. brucei* proliferation at 50 µM. Roscovitine is an inhibitor of cyclin-dependent kinases as well as mammalian PdxKs, although in the case of PdxK, it binds in the PL site rather than the ATP binding site (Bach *et al*., 2005; Tang *et al*., 2005). Roscovitine inhibited *T. brucei* proliferation with an EC_50_ of 7.6 ± 0.4 µM (data not shown). However, this value varied less than twofold when the inhibitor was tested in the presence of excess pyridoxal (HMI9T) giving an EC_50_ of 8.3 ± 0.7 µM, or against the conditional null mutant giving an EC_50_ of 5.3 ± 1.1 µM. Hence, other targets within the cell appear to be more significant than *Tb*PdxK.

**Fig. 5:**
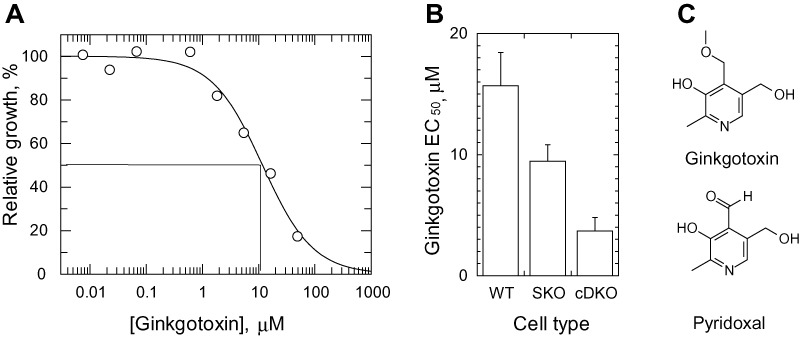
Effect of ginkgotoxin on *T. brucei* cells. A. An example of an EC_50_ determination is shown. Microplates were seeded with 1 × 10^3^ cells ml^−1^ and incubated in the presence of Ginkgotoxin (50 µM to 23 nM) for 3 days. Cell proliferation was assayed with resazurin. EC_50_ values were determined on three separate occasions. B. Mean EC_50_ values, weighted to the standard error, for the parental ‘single marker’ line (WT) are compared with those for the single-allele *PdxK* knockout (SKO) and the conditional null mutant.

Ginkgotoxin [4′-O-methylpyridoxine – a substrate analogue and nanomolar inhibitor of human PdxK (Kastner *et al*., 2007)] inhibited *T. brucei* proliferation in PDM with an EC_50_ of 15.7 ± 2.7 µM (Fig. 5A). In the presence of excess pyridoxal, inhibition was completely abrogated (inactive at 50 µM) suggesting either on-target activity against pyridoxal kinase or competition with B_6_ vitamers for uptake into the cell. The latter does not appear to be a significant factor because the EC_50_ showed a dose-relationship with the level of *Tb*PdxK available in the cell (Fig. 5B). In the single-allele deletion cell line, the EC_50_ was reduced 1.7-fold from 15.7 ± 2.7 µM to 9.4 ± 1.4 µM and in the cDKO (where tetracycline was removed at the start of ginkgotoxin incubation) the EC_50_ (3.7 ± 1.1 µM) was reduced fourfold compared with WT cells.

The low *K*_m_^app^ of *Tb*PdxK with respect to pyridoxal in our system translated to a low dynamic range when assaying at S = *K*_m_. It was therefore not practical to study the effect of these three inhibitors on the recombinant enzyme.

### Structural characterization

**Fig. 6:**
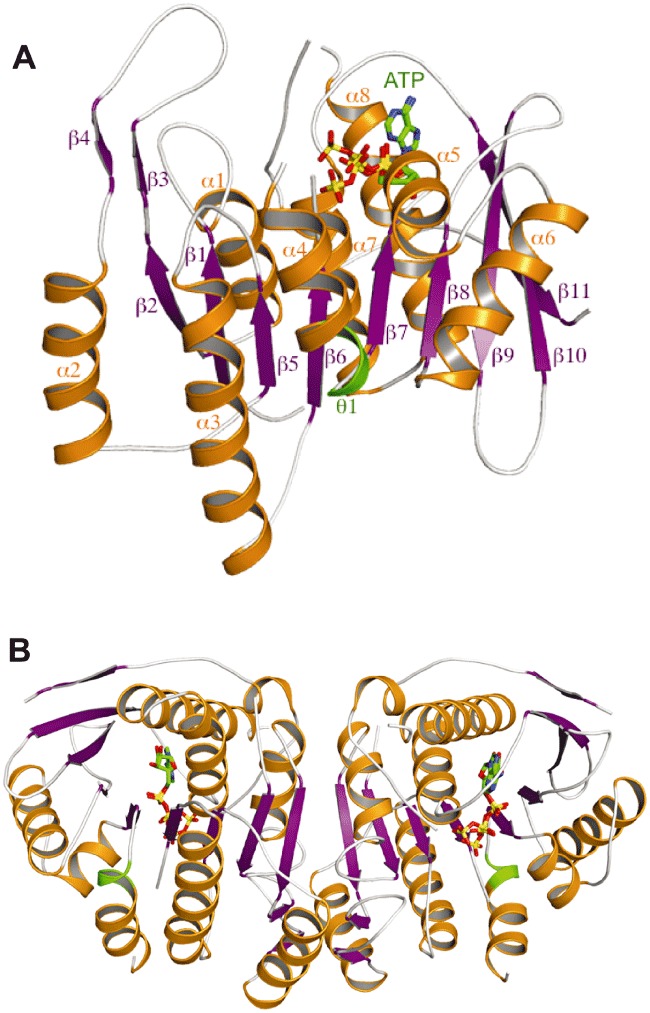
Structure of *T. brucei* PdxK in complex with ATP. A. *Tb*PdxK monomeric subunit with secondary structure annotated according to Fig. 7. α-Helices are in orange, β-strands are in magenta, the 3_10_-helix is in green and ATP is shown with carbon atoms in green, nitrogen atoms in blue, oxygen atoms in red, and phosphorus atoms in yellow. Figure produced using PyMOL (DeLano, 2005). B. The dimeric assembly of *Tb*PdxK annotated as in panel A.

Recombinant *Tb*PdxK was crystallized, diffraction data obtained to 2.0 Å and the structure solved using molecular replacement using the coordinates of the human enzyme (PDB 2YXT) as a starting model. The resulting structure revealed that *Tb*PdxK has a typical ribokinase central core of β-sheets surrounded by α-helices (Mathews *et al*., 1998; Sigrell *et al*., 1998; Campobasso *et al*., 2000; Schumacher *et al*., 2000; Cheng *et al*., 2002; Li *et al*., 2002). Although only a monomer is found in the asymmetric unit of our model (PDB code 3ZS7), it exists as a dimer in solution (Fig. 3B) and the crystallographic symmetry forms the active dimer. The monomer subunit and dimeric assembly are shown in Fig. 6. The secondary structure of each monomer comprises a 6-stranded parallel central β-sheet (strands 1, 2, 5, 6, 7, 8), a 2-stranded anti-parallel β-sheet (strands 3 and 4), and a 3-stranded anti-parallel β-sheet (strands 9, 10, 11). These are flanked by four α-helices on one side and 3 on the other. A short 3_10_-helix follows α4. Two loops (120–129, and 266–274) are missing from the model due to their flexibility and resulting lack of electron density, and several amino acids had to be truncated to alanine residues in the model as a result of side-chain flexibility (Lys99, Lys130, Glu131, Arg188, Arg223, Glu265 and Ser275).

**Fig. 7:**
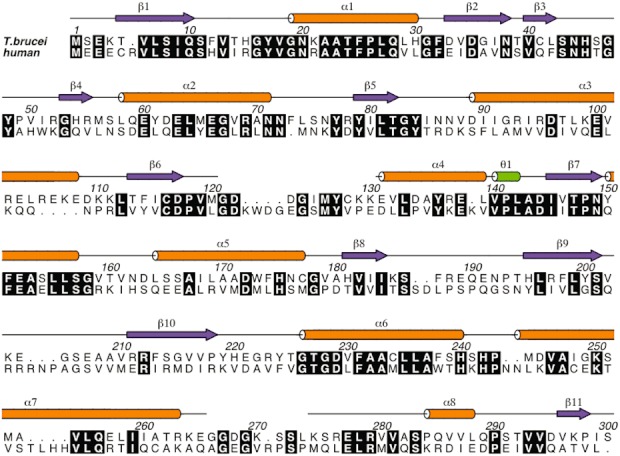
Sequence alignment of *Tb*PdxK with human PdxK. Conserved residues are highlighted in black. Sequences are numbered according to *Tb*PdxK. The secondary structure of *Tb*PdxK is indicated on the top line with α-helices in orange, β-strands in magenta, and 3_10_-helices in green. Figure produced using ALINE (Bond and Schuttelkopf, 2009).

Comparison between the human [2YXU (Musayev *et al*., 2007); 3KEU (Gandhi *et al*., 2009)] and *T. brucei* PdxK monomers, which have a sequence identity of 39%, shows an r.m.s.d. of 1.4 Å. The core secondary structure overlays well, as would be expected with such high sequence identity. The main areas of difference are the loops around the outside of structure. Loop β8–β9 (residues 185–196) is two residues shorter in the *T. brucei* PdxK. Loop α3–β6 is extended by four residues in the *T. brucei* structure, while β9–β10 is three residues shorter (Fig. 7).

### ATP binding site structure

**Fig. 8:**
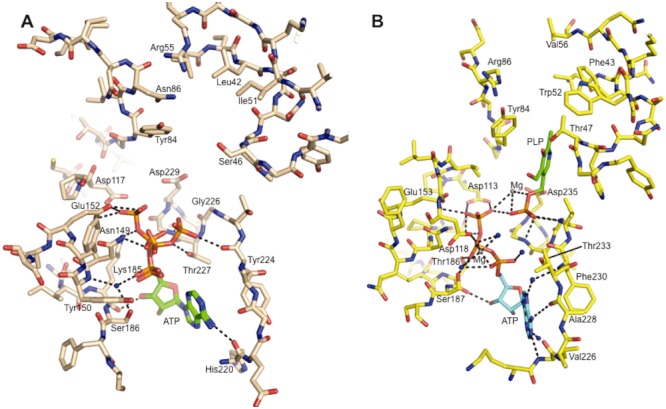
Important residues in and around the substrate and cofactor binding sites of parasite and human PdxK. A. *T. brucei* PdxK. B. Human PdxK (Gandhi *et al*., 2009). Oxygen atoms are red, nitrogen atoms blue, phosphorus atoms orange, and magnesium grey. Hydrogen bonds are shown as black dotted lines. Important residues, ATP, and pyridoxal-5′-phosphate (PLP) are labelled.

The crystal structure of *Tb*PdxK shows that the ATP takes on two alternate conformations in this crystal form (Fig. 8). The two modes of ATP binding resemble those seen in human and sheep [1LHR (Li *et al*., 2002)] PdxK. Compared with the human and sheep ATP-bound form, the adenine ring is rotated through 90° resulting in different protein contacts. The *Tb*PdxK adenine amine interacts with His220 carbonyl while in the human and sheep enzymes the interactions are with Val226, Ala228, and Phe230 backbone atoms (the latter via a water molecule). The ribose interacts with backbone carbonyls from equivalent residues in human and sheep PdxK. The phosphates adopt distinct conformations in the two structures. In *Tb*PdxK, the α-phosphate interacts via a water molecule with Tyr150 and Ser186. In human PdxK, the interaction is directly with Thr233 on the opposite side of the binding site. The β-phosphate of *Tb*PdxK interacts with Asn149 and Lys185, and with Thr186 in human PdxK. In the two different ATP binding modes of *Tb*PdxK, the γ-phosphates make different interactions. In one conformation, the γ-phosphate interacts with Asp117 and Glu152, as seen in human and *E. coli* PdxK (Safo *et al*., 2006). In the second conformation, the interactions are with Thr227, as in sheep PdxK, and backbone atoms of Tyr224 and Gly228. The conserved GTGD ribokinase motif (Safo *et al*., 2006) is present in the *Tb*PdxK amino acid sequence (residues 226–229; Fig. 7), and is seen in this structure to bind the γ-phosphate as in sheep PdxK, rather than the α-phosphate as seen for human and *E. coli*. In the alternative ATP conformation of *Tb*PdxK, the GTGD motif plays no role in ATP binding. However, in both conformations ATP is some distance from the potential pyridoxal binding site and as a result, either cofactor, substrate, or both would have to move a considerable distance to interact (Safo *et al*., 2006).

### Active site structure

Loop β6–α4 (residues 120–130) is missing in the *Tb*PdxK model due to disorder (Fig. 6). This loop is flexible and is seen to adopt a variety of conformations in different ribokinase structures (Mathews *et al*., 1998; Sigrell *et al*., 1998; Campobasso *et al*., 2000; Schumacher *et al*., 2000; Cheng *et al*., 2002; Li *et al*., 2002; Tang *et al*., 2005; Safo *et al*., 2006). In *Tb*PdxK, loop β6–α4 is situated adjacent to the substrate binding site at one end of the ATP binding site. The loop in the human PdxK is four residues longer than that of *Tb*PdxK. In *Tb*PdxK the equivalent loop would be 10 residues, and the sequence alignment (Fig. 7) shows that only five out of these 10 residues are conserved. Of the remaining five residues, Asp123 is substituted by a glutamate in human PdxK, Ile125 by serine, Cys128 by valine, Lys129 by proline, and Lys130 by glutamate.

Although the majority of residues in close proximity to the pyridoxal binding site, identified in PDB model 3KEU (Gandhi *et al*., 2009), are identical or conserved between *T. brucei* and human, including residues in loops β1–α1, β3–β4 and β5–α3, there are several differences as shown in Fig. 8. Leu42 in *Tb*PdxK (Fig. 8A) is replaced by Phe43 in human (Fig. 8B), though both occupy similar positions, and similarly Ile51 is replaced by Trp52. Ser46 is replaced by Thr47, with Oγ directed at the predicted substrate binding site. Though Arg55 is a valine in the human PdxK sequence, the Arg side-chain is around 6.5 Å from the expected binding site. *Tb*PdxK Asn86 is substituted by Arg86 in human and stacks above Tyr84, around 3.5 Å from the binding site, 7 Å closer than the human Arg86 side-chain. The differences outlined above in the pyridoxal binding site of *Tb*PdxK and human PdxK, in addition to the sequence differences in the unresolved flexible loop β6–α4 and ATP binding site may be exploitable for the design of specific inhibitors against *Tb*PdxK. Of particular interest are the positions and properties of Arg55 and Asn86.

### Conclusion

The genetic studies presented here clearly demonstrate that pyridoxal kinase activity is essential for growth and survival of *Trypanosoma brucei in vitro* and *in vivo*. The on-target inhibition by gingkotoxin demonstrates that the enzyme is druggable in the whole cell and therefore serves as proof of concept in developing drug leads against *Tb*PdxK. Our screening and assay work makes development of a high throughput screening platform possible. The vitamin B_6_-depleted medium developed here will be applicable for whole-cell screening of inhibitors. Given the structural information elucidated here, *Tb*PdxK is now well placed to enter the drug discovery pipeline.

## Experimental procedures

### Chemicals and enzymes

All chemicals were obtained from Sigma Aldrich (Gillingham, UK) unless otherwise stated. Ginkgotoxin was a generous gift from Dr Thomas Hemscheidt at the University of Hawaii. Roscovitine was obtained from Calbiochem. IMDM was obtained from PAA labs (Yeovil, UK). Polymerase and restriction enzymes were obtained from Promega (Southampton, UK).

### Trypanosome culture

*Trypanosoma brucei* bloodstream form ‘single marker’ S427 (T7RPOL TETR NEO) and cell lines derived from them were cultured in HMI9T medium (Greig *et al*., 2009) or vitamin B_6_-depleted medium (PDM) as indicated. PDM is equivalent to HMI9T without the Serum+™ additive (HMI11, Hirumi and Hirumi, 1989) and the IMDM base medium is assembled in-house without vitamin B_6_. G418 at 2.5 µg ml^−1^ is used to maintain expression of T7 RNA polymerase and the tetracycline repressor protein (Wirtz *et al*., 1999). HMI9T and PDM contain 16.9 µM and ∼ 20 nM vitamin B_6_ respectively (see *Results and discussion*). Cells were grown at 37°C in 5% CO_2_. Cell density was determined using a haemocytometer (Neubauer).

### Oligonucleotides and sequencing

Primers obtained from Thermo Scientific (Ulm, Germany) were designed using the ORF and flanking region of *PdxK* (*T. brucei* strain 927, GeneDB Tb927.6.2740). All constructs were verified by sequencing.

### Sequencing of the *TbPdxK* ORF

The ORF of *PdxK* together with approximately 200 bp upstream and downstream adjacent sequence was amplified from ‘single marker’ genomic DNA in three independent PCRs using Pfu or *Taq* polymerases. Each of the products was sequenced following cloning into pCR-BluntII TOPO or pCR2.1-TOPO (Invitrogen) and plasmid purification using the Qiaprep spin miniprep DNA kit (Qiagen). The consensus sequence was used for confirming error-free amplification of the ORF for expression constructs.

### Generation of knockout and regulated expression constructs

The 5′-sequence (497 bp) of and 3′-sequence (500 bp) immediately flanking the ORF of *TbPdxK* were amplified from *T. brucei* S427 genomic DNA by PCR using GoTaq™ polymerase and subsequently cloned into pGem5ZF. PCR primers used in these and subsequent experiments are given in Table S1. BamHI and HindIII sites introduced during PCR were used to insert the selectable markers *PAC* or *HYG* between the flanking sequences to produce two ‘knockout’ constructs. NotI restriction sites also added during PCR allowed the entire assembly of flanking regions and selectable marker to be excised intact from the construct to produce linear DNA for electroporation.

To generate a *T. brucei* regulated expression construct, the ORF of *PdxK* was amplified by PCR using Pfu polymerase and cloned directly into pCR-BluntII TOPO (Invitrogen). The 5′ primer used for PCR contained a HindIII restriction site before the start codon. The 3′ primer contained a BamHI restriction site immediately following the stop codon. Following triplicate sequencing throughout the ORF, it was subcloned into pLew100 (Wirtz *et al*., 1999) using the HindIII and BamHI sites introduced during the PCR.

To generate a recombinant expression construct for *E. coli*, an identical strategy was employed as above. In this case the 5′ primer used for PCR contained an NdeI restriction site before the start codon. The 3′ primer contained a BamHI restriction site following the stop codon.

### Generation of *T. brucei* mutants

Knockout and regulated expression constructs were prepared using the HiSpeed Plasmid Midi Kit (Qiagen). Constructs were linearized with NotI, ethanol precipitated and redissolved in sterile water at a final concentration of 1 µg ml^−1^. *T. brucei* were cultured in HMI9T before and during transformation by electroporation and Southern blotting analysis. Cells were electroporated with 10 µg of DNA using the Human T cell Nucleofector kit according to manufacturer's instructions and programme X-001 of the Nucleofector II electroporator (Amaxa, Cologne, Germany) (Burkard *et al*., 2007).

The single marker cell line was transfected with the HYG knockout construct and cells selected in the presence of 4.0 µg ml^−1^ hygromycin. This single knockout line was transfected with the regulated expression construct to produce a conditional single knockout (cSKO) line selected for transfectants with 6 µg ml^−1^ phleomycin. The cSKO cell line was then continuously cultured with 0.5 µg ml^−1^ tetracycline to induce expression of PdxK. Transfection of the cSKO line with the PAC knockout construct followed by selection with 0.1 µg ml^−1^ puromycin yielded the cDKO null mutant. The cDKO line was maintained in the presence of G418, hygromycin, puromycin, phleomycin and tetracycline at the concentrations indicated above. Tetracycline was removed from cells as indicated in the text by washing in phosphate buffer saline containing 25 mM glucose.

### Southern blot analysis of transgenic *T. brucei* cell lines

Correct integration of the knockout and regulated expression constructs were confirmed by digestion of 5 µg of gDNA with BglII and subsequent Southern blot analysis. Samples were electrophoresed in 0.8% (w/v) agarose gels and transferred to nylon membrane (Roche) using a Vacugene blotter (GE Healthcare) according to manufacturer's instructions. DNA probes specific for the PdxK ORF and 5′ UTR were produced using GoTaq™ polymerase and the PCR DIG (digoxigenin) Probe Synthesis Mix (part of the PCR DIG Probe Synthesis Kit from Roche). Probes were hybridized in DIG EasyHyb buffer (Roche) and washed and detected using the DIG wash and block buffer set (Roche) both according to kit instructions.

### Immunoblotting

Polyclonal antisera against *Tb*PdxK were raised in adult male Wistar rats. An initial injection of 100 µg of purified antigen, emulsified in complete Freund's adjuvant, was followed by two identical booster injections of antigen emulsified in Freund's incomplete adjuvant at 2-week intervals. Polyclonal antiserum against *T. brucei* tryparedoxin (TryX) was previously prepared in the same manner (Konig *et al*., 2011). Cells (5 × 10^6^) were harvested by centrifugation (800 *g*, 10 min, 25°C); the supernatant was aspirated leaving approximately 10 µl. Pellets were resuspended in the remaining supernatant; 10 µl Laemmli buffer (Laemmli, 1970) was added and the samples boiled for 10 min. Samples were stored at −20°C until the end of time-course experiments. Samples were then separated by SDS-PAGE and subsequently transferred onto Protrans nitrocellulose membrane (Whatman). After blocking with 5% (w/v) dried skimmed milk in PBS + 0.05% (v/v) Tween-20 at room temperature for 1 h, the blocking solution was replaced and incubated overnight. All remaining washes and incubations were carried out in 1% (w/v) dried skimmed milk in PBS + 0.05% (v/v) Tween-20. Blots were washed and incubated with *T. brucei* PdxK polyclonal antiserum (1/1000 dilution) or *T. brucei* TryX polyclonal antiserum at room temperature for 1 h. Blots were washed and then incubated with a secondary rabbit anti-rat (IgG) antibody (Dako; 1/1000 dilution). The immunoblots were subsequently developed using the ECL® (enhanced chemiluminescence) system from Amersham Biosciences.

### EC_50_ determination of PdxK inhibitors against *T. brucei* cells

Ginkgotoxin, roscovitine and 4-deoxypyridoxine were tested against the single marker parental cell line, SKO and cDKO cells in HMI9T and PDM. Assays were carried out in 96-well plates using a resazurin-based assay as described previously (Raz *et al*., 1997; Jones *et al*., 2010). EC_50_ values were determined in triplicate using GraFit software (Erithacus Software) with a non-linear 3-parameter curve fit. Initial assay conditions were as follows, 0.5% (v/v) dimethylsulphoxide (DMSO); 1 × 10^3^ ml^−1^ cells; 0–50 µM inhibitor. Values are presented as means weighted to the standard error.

### *In vivo* studies

Wild-type and transgenic bloodstream *T. brucei* parasites were cultured in the absence of selectable drugs for 24 h prior to infection of mice. During this time, cDKO cells were grown in the presence or absence of 1 µg ml^−1^ tetracycline. These parasites were then used to infect groups of five mice (dosed with and without doxycycline respectively) by a single intraperitoneal injection of 10^4^ parasites in 0.2 ml of HMI9T medium. The ‘plus’ doxycycline group of animals were dosed with doxycycline in their drinking water [0.2 mg ml^−1^ in a 5% (w/v) sucrose solution] for 5 days prior to infection and freshly prepared every second day for the duration of the experiment. Animals were inspected daily for clinical signs of infection and wet smears of tail blood were examined microscopically. Parasitaemia was determined using a Neubauer haemocytometer. Mice that exceeded a parasitaemia > 10^8^ ml^−1^ were humanely killed, since prior experience indicated that animals would succumb to an overwhelming infection by the following day (Sienkiewicz *et al*., 2008).

### Expression and purification of recombinant protein from *E. coli*

The pET3a expression construct was transformed into BL21(DE3) cells (Stratagene) by heat shock. A single colony was used to inoculate 10 ml Luria–Bertani medium containing 50 mg ml^−1^ ampicillin and the culture grown overnight at 37°C. The overnight culture was diluted 200-fold to inoculate a 1 l culture. Expression cultures were grown at 37°C until the OD_600_ reached 0.6. Recombinant expression was induced with 0.4 mM IPTG, and expressed for a further 18 h at 20°C.

Bacteria were collected by centrifugation (5000 *g*, 25 min) and resuspended in 25 mM Tris pH 7.5 containing 100 mM NaCl, a few grains of DNAse I and Complete EDTA-free protease inhibitor cocktail (Roche). Bacteria were lysed in a One Shot cell disruptor (Constant Systems), the extract clarified by centrifugation (40 000 *g*, 20 min) and the supernatant diluted to 25 mM NaCl.

*Tb*PdxK was purified using a HiTrap™ Q HP anion exchange column (GE Healthcare). The column was eluted with a gradient of 25 mM to 1 M NaCl using an Akta Purifier monitoring at 280 nm. PdxK-containing fractions eluted at ∼ 65 mM and ∼ 105 mM NaCl. Fractions were pooled based on SDS-PAGE results. The fractions eluting at 105 mM NaCl contained less than 20% of the PdxK activity (see assay details below) and contained contaminating protein bands (data not shown). Fractions eluting at 65 mM NaCl were pooled and concentrated using a 30 kDa PES Vivaspin concentrator (Sartorius).

For gel filtration, the material from anion exchange was dialysed into 25 mM Tris pH 7.5 containing 100 mM NaCl and applied to a Superdex 75 column (26/60, GE Healthcare). Elution of the column in the same buffer was monitored at 280 nm using an Akta Purifier. Relative molecular mass (M_r)_ was estimated using protein gel filtration standards (Bio-Rad) on a plot of elution volume versus Log M_r_. The recombinant enzyme was stable for more than 18 months when stored at −20°C in 50% v/v glycerol.

Protein concentrations were determined using the Bradford assay (Bradford, 1976). SDS-PAGE was carried out with the NuPAGE system (Invitrogen) using 4–12% Bis-Tris gels and staining with InstantBlue (Expedeon). Identity by peptide mass fingerprinting was carried out by the Fingerprints proteomics facility (College of Life Sciences, University of Dundee, Scotland, http://proteomics.lifesci.dundee.ac.uk).

### Kinetic characterization by direct assay (388 nm)

*Tb*PdxK was assayed spectrophotometrically by monitoring formation of PLP at 388 nm as previously described (Scott and Phillips, 1997) using either 70 mM phosphate pH 7.0 or 100 mM HEPES pH 7.4 as indicated in the text. ATP (1.5 mM) was used in all assays. Reactions were carried out at 37°C and 27°C as indicated in 1 ml acrylic cuvettes and changes in absorbance monitored with a UV-1601 PC spectrophotometer (Shimadzu). 1 U of enzyme activity equals 1 µmol PLP formed per min.

*K*_m_^app^ values with respect to substrates were determined in three independent experiments using Grafit 5 (Erithacus Software) and a mean weighted to the standard error calculated (Jones *et al*., 2010). Assays contained 10 mU ml^−1^ PdxK and were initiated with PL which was varied from 50 µM to 1.5 µM. *K*_m_^app^ determinations in phosphate buffer contained 150 µM ZnCl_2_, those in HEPES buffer contained 250 µM MgCl_2_ or 150 µM ZnCl_2_. Data were fitted to the Michaelis–Menten equation or, where appropriate, to the following equation for high substrate inhibition:


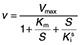


where *K*_i_^s^ is the inhibition constant for substrate.

Preference for divalent cations was measured in 100 mM HEPES pH 7.4 containing 300 µM PL and 10 mU ml^−1^ PdxK. Several divalent cations as indicated in the text were added at 100 µM, and reaction rates measured in duplicate reactions. MgCl_2_ and MnCl_2_ were titrated from 1 mM to 5 µM in seven twofold dilutions, and the reaction rates measured in duplicate reactions.

### Determination of absorption coefficients

Molar absorption coefficients for PL and PLP have been previously determined at pH 7.0 as 200 (at 390 nm) and 4900 (at 388 nm) respectively. Molar absorption coefficients for PM and PMP have been determined at pH 7.0 as 7700 and 8300 respectively (at 325 nm in both cases) (Peterson and Sober, 1954). Solutions of all four vitamers were made up at arbitrary concentrations in water. For each vitamer, samples were diluted 100-fold into 1 ml acrylic cuvettes, three containing 100 mM potassium phosphate, pH 7.0 and three containing 100 mM potassium phosphate pH 7.4. The absorbance of samples at pH 7.0 was measured at 390 nm, 388 nm and 325 nm as indicated above, and the published absorbance coefficients used to calculate the molar concentration of the stock solutions by Beer–Lambert's law. The absorbance of samples at pH 7.4 was measured at 340 nm and used with the calculated molar concentrations to determine the molar absorbance coefficients under these conditions. These values were used to determine the net absorbance coefficients for the NADH coupled assay. No correction was required for ginkgotoxin or PN.

### Kinetic characterization by coupled assay (340 nm)

In order to investigate the use of other substrates, the production of ADP by *Tb*PdxK was coupled to consumption of NADH using pyruvate kinase and lactate dehydrogenase. Each assay contained 100 mM HEPES pH 7.4, 1.5 mM ATP, 1 mM MgSO_4_, 0.2 mM NADH, 1 mM phosphoenolpyruvate, 10 mM KCl, 2% (v/v) glycerol, 2.5% (v/v) DMSO and pyruvate kinase/lactate dehydrogenase mix (approximately 1.6 and 2.3 U ml^−1^ respectively) from rabbit muscle (Sigma). Assays were carried out at 27°C in 1 ml acrylic cuvettes and changes in absorbance at 340 nm monitored with a UV-1601 PC spectrophotometer (Shimadzu). Rates were determined using the appropriate extinction coefficient given in Table S2.

*K*_m_^app^ values with respect to PL, PM, PN and ginkgotoxin were determined in three independent experiments and a mean weighted to the standard error calculated (Jones *et al*., 2010). Assays contained 10 mU ml^−1^ of *Tb*PdxK and were initiated with the relevant substrate which was varied from 250 µM to 3 µM.

### Crystallization

Purified protein was concentrated to 6 mg ml^−1^. ATP (1 mM) and MgCl_2_ (1 mM) were added to the protein, which was then filtered through a 0.1 µM spin filter. Initial crystallization conditions were identified using the JCSG sparse matrix screen (Jena Biosciences). Crystals grew at room temperature in 20% polyethylene glycol (PEG) 3350 and 0.2 M (NH_4_)_2_Cl using the sitting drop method with 0.75 µl protein and 0.75 µl precipitant. These crystals were improved using 16% PEG 4000 and 0.2 M (NH_4_)_2_Cl with 1 µl + 1 µl hanging drops, and were further optimized by addition of 0.25 µl of 0.1 M NaF from the Hampton Research additive screen. This yielded crystals in 1–2 days belonging to space group C222_1_ with unit cell dimensions a = 69 Å, b = 80 Å, c = 106 Å.

### Data collection, structure solution and refinement

For data collection, a single crystal was cryoprotected in artificial mother liquor substituted with 30% PEG 3350 and flash frozen in a stream of nitrogen at 100 K. The data were collected using a Rigaku MicroMax 007 X-ray source coupled to an RAXIS IV++ image plate detector. Data were processed and scaled using the XDS package (Kabsch, 1993) and details can be seen in Table S3. The structure was solved by molecular replacement with MOLREP (Vagin and Teplyakov, 1997) using PDB entry 2YXT (Musayev *et al*., 2007) as a starting model. The resulting model from MOLREP then underwent several rounds of manual model building using COOT (Emsley and Cowtan, 2004) and rigid body and positional refinement using REFMAC5 (Murshudov *et al*., 1997). Water molecules and ATP were added and refined. The resulting model has R_work_/R_free_ values of 22%/26% respectively. Some residues had to be omitted due to weak electron density. Further refinement statistics can be seen in Table S3.
