# Supplementary material for: Chemical, genetic and structural assessment of pyridoxal kinase as a drug target in the African trypanosome
Source: Mol Microbiol. 2012 Aug 16;86(1):51–64. doi: 10.1111/j.1365-2958.2012.08189.x (PMC3470933; doi:10.1111/j.1365-2958.2012.08189.x)
Supplement: Supplementary file 1 [file mmi0086-0051-SD1.doc]

**SUPPLEMENTARY DATA**

**Chemical, genetic and structural assessment of pyridoxal kinase as a drug target in the African trypanosome**

Deuan C. Jones, Magnus Alphey, Susan Wyllie and Alan H. Fairlamb

**Fig. S1** Growth characteristics and biochemical analysis of cDKO cells in vitro.

The growth of the cDKO cell line in HMI9T medium was assessed in the presence (open circles) and absence of tetracycline (closed circles).

**Fig. S2** Effect of vitamer supplementation on growth of cDKO cells in vitro.

The cDKO cell line was grown in the presence or absence of tetracycline and vitamers supplemented at 1.34 μM as indicated. Cells were grown in PDM for two days, diluted 100-fold and grown for a further two days. The mean and standard deviation of two experiments is shown.

**Table S1 PCR primers**

Upper case letters are used to indicate nucleotides complementary to *T. brucei* sequences. Restriction endonuclease sites are underlined.

| **PCR primer sequence** | **Size of product (bp)** | **PCR product details** |
| --- | --- | --- |
| TTTCTATTGGAAAACAAATAACC | 1272 | ORF + 200 bp flanking regions used for sequencing. |
| TTCGTTTTGTATGCGATTTACC |
| ttagcagcggccgcTTTACAGGGAACCGATTTAGC | 497 | 5’UTR used to generate knockout construct. |
| tggacggtttaaacctaagcgaagcttTATGTGTCCTTGTTTTTCAC |
| cgcttaggtttaaaccgtccaggatccGAATTAAATGCAGTCTGTCAAG | 500 | 3’UTR used to generate knockout construct. |
| tagtaagcggccgcCAGATCTGAATATCCAGTTCC |
| tcagtaaagcttACACACATTTCGTGCGAAGG | 981 | ORF and short UTR used to produce pLew100-based regulated expression construct. |
| ggagaaggatccGGTGGACTTGACAGACTGC |
| ctggatcatATGTCAGAGAAGACGGTTTTAT | 903 | ORF used to produce pET3a-based recombinant expression construct. |
| gcgtaaggatccTCATGAAATTGGTTTAACGTC |

**Table S2 Molar absorption coefficients of vitamers of vitamin B_6_**

| **Vitamer** | **Coefficients at pH 7.0**  **(M^-1^ cm^-1^)^a^** | **Calculated ε_340nm_ for pH 7.4^b^**  **(M^-1^ cm^-1^)** | **Δε_340nm_ for NADH coupled assay**  **(M^-1^ cm^-1^)** |
| --- | --- | --- | --- |
| **Pyridoxal (PL)** | ε_390nm_ = 200 | 1535 ± 49 | 5500 |
| **Pyridoxal phosphate (PLP)** | ε_388nm_ = 4900 | 2205 ± 65 |
| **Pyridoxamine (PM)** | ε_325nm_ = 7700 | 4196 ± 110 | 5825 |
| **PMP** | ε_325nm_ = 8300 | 4591 ± 173 |

^a^ Data from (Peterson and Sober, 1954)

^b^ Mean ± standard deviation (n=3)

**Table S3 Refinement statistics for *Tb*PdxK**

|  | - |
| --- | --- |
| **Resolution range (Å)** | 19.7 – 2.0 |
| **Total observations** | 90864 |
| **Unique reflections** | 18739 |
| **Redundancy** | 4.8 (4.8) |
| **Completeness** | 92.2 (84.7) |
| **I/σI** | 23.7 (5.2) |
| **R_merge_** | 5.5 (26.6) |
|  |  |
| **R** | 21.9 (25.5) |
| **R_free_** | 26.2 (29.9 ) |
| **Reflections used** | 18420 |
| **Test set** | 970 |
| **rmsd bond length** | 0.011 |
| **rmsd bond angles** | 1.47 |
|  |  |
|  |  |
| **Ramachandran** |  |
| **Allowed regions (%)** | 99.2 |

Supplementary Reference

Peterson,E.A., and Sober,H.A. (1954) Preparation of crystalline phosphorylated derivatives of vitamin B6. *J Am Chem Soc* **76**: 169-175.
